# Effect of the Application of a Coating Native Potato Starch/Nopal Mucilage/Pectin on Physicochemical and Physiological Properties during Storage of Fuerte and Hass Avocado (*Persea americana*)

**DOI:** 10.3390/polym14163421

**Published:** 2022-08-21

**Authors:** David Choque-Quispe, Yasmine Diaz-Barrera, Aydeé M. Solano-Reynoso, Yudith Choque-Quispe, Betsy S. Ramos-Pacheco, Carlos A. Ligarda-Samanez, Diego E. Peralta-Guevara, Edgar L. Martínez-Huamán, John Peter Aguirre Landa, Odilon Correa-Cuba, Henrry W. Agreda Cerna, Mery Luz Masco-Arriola, Washington Julio Lechuga-Canal, Julio C. Loayza-Céspedes, Genaro Julio Álvarez-López

**Affiliations:** 1 Water Analysis and Control Research Laboratory, Universidad Nacional José María Arguedas, Andahuaylas 03701, Peru; 2 Department of Agroindustrial Engineering, Universidad Nacional José María Arguedas, Andahuaylas 03701, Peru; 3 Research Group in the Development of Advanced Materials for Water and Food Treatment, Universidad Nacional José María Arguedas, Andahuaylas 03701, Peru; 4 Escuela de Posgrado, Universidad de San Antonio Abad del Cusco, Cusco 08000, Peru; 5 Department of Environmental Engineering, Universidad Tecnológica de los Andes, Andahuaylas 03701, Peru; 6 Department of Environmental Engineering, Universidad Nacional José María Arguedas, Andahuaylas 03701, Peru; 7 Food Nanotechnology Research Laboratory, Universidad Nacional José María Arguedas, Andahuaylas 03701, Peru; 8 Department of Education and Humanities, Universidad Nacional José María Arguedas, Andahuaylas 03701, Peru; 9 Department of Business Administration, Universidad Nacional José María Arguedas, Andahuaylas 03701, Peru; 10 Department of Basic Sciences, Universidad Nacional José María Arguedas, Andahuaylas 03701, Peru; 11 Department of Chemical Engineering, Universidad Nacional de San Antonio Abad del Cusco, Cusco 08000, Peru; 12 Departamento de Ingeniería Agropecuaria, Universidad Nacional de San Antonio Abad del Cusco, Andahuaylas 03701, Peru; 13 Law and Humanities Faculty, Universidad Continental, Cusco 08000, Peru

**Keywords:** coating, fuerte avocado, hass avocado, pectin, physicochemical properties, physiological properties, storage

## Abstract

The avocado fruit is an agro-industrial product with high export demand in Peru due to its sensory and nutritional qualities, which can be affected during storage. The study aimed to evaluate the effect of the application of a coating formulated with potato starch (*Solanum tuberosum* ssp andigena), nopal mucilage (*Opuntia ficus* indica), and pectin on the physicochemical and physiological properties during the storage of Fuerte and Hass avocados. Samples were taken in their harvest state from the plantation in “Occobamba”, which is cultivated by the Avocado Producers Association in Chincheros, Apurímac, Peru. Physicochemical properties (titratable acidity, pH, total soluble solids) and physiological properties (weight loss, firmness, and color L* a* b*) were determined during 20 days of storage at 20 °C. The elaborated films present high transparency and low *a_w_* values. In the coated avocado of the Hass and Fuerte varieties, acidity and total soluble solids decreased significantly (*p*-value < 0.05) during the storage time. Weight loss and firmness of coated fruits decrease to a lesser extent. Luminosity L*, color index, and color variation showed better attributes for the coated samples. The use of coatings made with potato starch, nopal mucilage, and pectin allows the physicochemical and physiological properties of avocado fruits to be maintained for a longer time during storage.

## 1. Introduction

Currently, avocado is a fruit that is experiencing growing exports, especially from Latin American countries such as Peru, Mexico, Colombia, Chile, and Brazil, because it is a fruit with a considerable amount of polyunsaturated fatty acids, bioactive compounds, and 7-carbon sugars, which give it a pleasant flavor and high nutritional value [1,2,3,4,5,6,7,8,9].

Avocado, like other climacteric fruits, undergoes rapid biochemical changes, showing in its appearance and in its composition (starch splitting to soluble sugars, pulp softening, color loss, and the appearance of aromas and odors characteristic of ripe fruit) [10,11,12].

The avocado should be firm and shiny at the time of selling, as well as healthy in appearance and free of microorganisms [13,14]. These characteristics can be maintained for extended periods under specific conditions through proper postharvest handling [1,12,15,16]; however, avocado fruits are threatened by the development of microorganisms and losses caused by the accelerated decomposition process, which affects the presentation and sensory quality of the product [9,17].

Different methods have been developed to maintain the internal and external qualities of the fruits, through the use of modified atmospheres, vacuum packaging, and coating with synthetic and biodegradable films [18,19,20]. In addition to extending shelf life, these methods improve stability and quality during storage, although they differ in cost and application technology [21,22].

The application of films or coatings on fruits is based on characteristics such as cost, availability, functional attributes, mechanical properties (tension and flexibility), optical properties (brightness and opacity), the barrier effect against gas flow, structural resistance to water and microorganisms, and their sensory acceptability [22,23,24,25].

On the other hand, the irrational use of synthetic polymers in food protection presents a worldwide problem, because the degradation of materials such as polyethylene and polypropylene is practically nil [23]. Therefore, there is a growing interest in the production of biodegradable and/or edible materials using natural polymers, such as proteins and polysaccharides [18,26,27], which are friendly to the environment and contribute to the circular economy.

In that sense, applying biodegradable coatings from renewable sources, such as lipids, polysaccharides, and proteins, reduces the rate of respiration; delays weight loss due to dehydration; prolongs firmness and pigmentation, preserving the quality; and extends the shelf life of perishable and/or minimally processed foods [15,26,28]. This translates into minimizing the economic loss due to the decrease in fruit weight during the physiological maturity of the fruit [29,30,31,32].

There are vegetable raw materials that do not require agricultural attention, such as nopal, or that are declared as discarded, as is the case of small-sized native potatoes (diameter less than 2 cm), which could be a potential source of use for the formulation of biodegradable films. To a great extent, these have not been characterized or used due to the varieties or ecotypes they present at different altitudes in which they develop.

There are numerous works on the use of coatings made from starches from different sources [23,31,33,34,35,36,37,38], however, it is possible to improve the properties of these coatings with the addition of other components such as nopal mucilage and pectin. For this reason, the present work intends to formulate biodegradable coatings based on native potato starch, nopal mucilage, and pectin, and characterize them through infrared analysis, DSC and TGA thermal stability, transparency, and measurement of water activity. Applying them to avocado fruits of the Hass and Fuerte avocado varieties in order to evaluate their physicochemical and chemical properties during storage.

## 2. Materials and Methods

### 2.1. Vegetal Material

The avocado fruits (*Persea americana*) of the Hass and Fuerte varieties in the state of harvest maturity with uniform pigmentation and without physical and biological damage were collected from the fields in “Ocobamba” of the Avocado Producers Association. With the coordinates: Latitude −13.483056° and Longitude −73.561111° at 3032 m altitude, from the Province of Chincheros, Apurímac, Peru.

### 2.2. Preparation of the Emulsion

Potato starch (*Solanum tuberosum* ssp andigena) of the Huamantanga variety was extracted by hydroextraction, and nopal mucilage (*Opuntia ficus* indica) was extracted by ethanolic precipitation [34,39].

The emulsions were prepared by adding the components (Table 1), taking as reference the formulations proposed by Choque-Quispe et al. [34], in the following order: potato starch solution (PS), nopal mucilage solution (NM), and citrus pectin grade 65 (PC) (Spectrum, New Brunswick, Canada), under continuous stirring until complete homogenization, then heated to 70 °C, and glycerol (G) (99.5%, Scharlau, Barcelona, Spain) under continuous agitation. The emulsion was allowed to cool in the environment until its application to the avocado fruits.

### 2.3. Determination of Transparency and Water Activity of Coatings

The emulsions were molded on glass plates at room temperature for 24 h, obtaining coating films. The films were conditioned in a quartz vial with a rectangular side, and the transmittance was read at 600 nm in a Thermo Fisher UV-Vis spectrophotometer, model Genesys 150 (Madison, WI, USA) [35]. Transparency was reported as the ratio between transmittance and thickness (nm/mm).

Samples of 1 cm × 1 cm were taken to a previously calibrated water activity (*a_w_*) determiner, Rotronic brand, model HygroPalm23-AW (Bassersdorf, Switzerland).

### 2.4. IR Analysis of the Coating

Tablets were prepared with 0.1% KBr (grade IR, Darmstadt, Germany). The readings were made in transmission mode in the FTIR spectrometer (Fourier transform infrared spectroscopy), Thermo Fisher, Nicolet IS50 model (Waltham, MA, USA), in a range of 4000 to 400 cm^−1^ with a resolution of 4 cm^−1^.

### 2.5. Thermal Analysis of the Coating

The thermal transition properties of the coatings were analyzed through a differential scanning calorimeter (DSC), TA Instruments brand, model DSC2500 (Waters TM, New Castle, DE, USA), under a nitrogen atmosphere (50 mL/min). Samples were sealed in an aluminum pan and scanned from 20 to 200 °C at a heating rate of 5 °C/min. The equipment was stabilized through a baseline run at analysis conditions for 1 h.

A thermogravimetric analysis (TGA) was applied to know the thermal stability of the coating. The samples were loaded in alumina crucibles, and taken to a TA Instruments brand equipment, model TGA550 (Waters TM, New Castle, DE, USA), in the range of 20 to 200 °C, heating rate of 10 °C/min, and nitrogen supply of 50 mL/min.

### 2.6. Coating Application

The emulsion was applied to avocado fruits of the Hass and Fuerte varieties through a conventional atomizer, verifying that the entire surface was covered, and it was allowed to dry at room temperature. Likewise, fruits without coating were considered as control of both varieties.

### 2.7. Determination of Physicochemical Properties

The titratable acidity of the pulp was determined as a percentage of citric acid, the pH, and total soluble solids of the coated avocado samples every two days, for 20 days at 20 °C of storage, according to the methodologies proposed by the AOAC [40].

### 2.8. Determination of Physiological Properties

Weight loss during storage was determined and expressed as a percent weight difference. Fruit firmness was measured in different parts of the fruit using a penetrometer [41].

The color characteristics of avocado peel during storage were measured using the Kónica-Minolta colorimeter, model CR-5 (Japan), luminosity L* was determined (0 = black and 100 = white), chroma a* (+a = red, −a = green), chroma b* (+b = yellow and −b = blue). The measurements were taken at previously defined points, and the average of the values was recorded [28,42].

Likewise, the color index (CI*) (Equation (1)) was determined, which allows color to be expressed in a single numerical data [43], whose interpretation is as follows:

- If CI* −40 to −20, colors range from blue-violet to deep green.
- If CI* −20 to −2, colors range from deep green to yellowish-green.
- If CI* −2 to +2, represents greenish-yellow.
- If CI* +2 to +20, colors range from pale yellow to deep orange.
- If CI* +20 to +40, colors range from deep orange to deep red.

(1) $$CI^{*}=\frac{a^{*}\times 1000}{L^{*}\times b^{*}}$$

In the same way, the color difference (ΔE*) was calculated with respect to the control sample (Equation (2)) [35].

(2) $$\Delta E^{*}=\sqrt{{\left(\Delta L^{*}\right)}^{2}+{\left(\Delta a^{*}\right)}^{2}+{\left(\Delta b^{*}\right)}^{2}}$$

ΔE* can be classified as very different (ΔE* > 3), different (1.5 < ΔE* < 3) and minimally different (ΔE* < 1.5) [44].

### 2.9. Statistical Analysis

A randomized complete block design was applied, and data were collected in triplicate and analyzed by two-factor ANOVA and Tukey’s multiple comparison at 5% significance through Statistica V12 software, demo mode.

## 3. Results and Discussion

### 3.1. Coating Characteristics

Film transparency is a very important sensory aspect during fruit coating [13,45]. Transmittance values of around 81% were reported for the elaborated coatings (*p*-value > 0.05) (Table 2). Values above 90% are considered transparent [35,46], suggesting that the films prepared could be considered suitable for avocado coating, whose transparency values are 6.939 and 7.332 nm/mm, being slightly higher for formulation F2 (*p*-value < 0.05).

The water activity (*a_w_*) allows us to indirectly know the hygroscopic capacity of the films due to the presence of active receptor sites for water molecules on their surface. In the same way, it allows to take criteria of microbiological aspects [42,47,48]. It was observed that the film with formulation F2 reported *a_w_* 0.404 ± 0.05 slightly lower than F1 (*p*-value < 0.05), that is, it would retain less water due to the lower presence of hydrophilic groups, preventing the water diffusion due to the film barrier [13,49,50], this would be due to the lower presence of pectin in the formulation.

**Figure 1:**
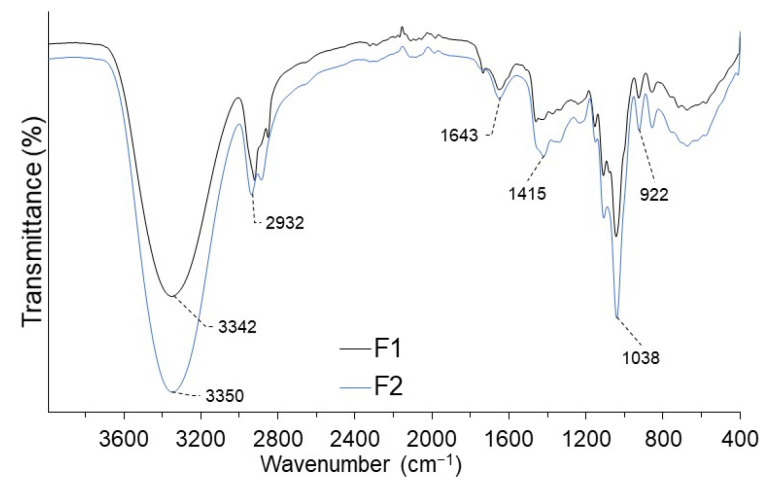
IR spectra for coating F1 and F2.

IR analysis revealed high-intensity peaks around 3350 cm^−1^, being higher for the F2 film (Figure 1). This is attributed to the presence of hydroxyl groups of carbohydrates and gums (basis of the structure of starch, nopal mucilage, and pectin) [37,38,39], which would allow it to retain higher moisture content. The spectrum around 2930 cm^−1^ is due to the stretching vibration of the carbohydrate methyl group. A small peak around 1640 cm^−1^ shows the presence of water adsorbed on the film, being slightly higher in F2, which confirms its ability to retain water on its surface, making it a slightly permeable material. Peaks around 1415, 1038, and 922 cm^−1^ evidence the presence of carboxyl, carbonyl, and methyl groups from polysaccharides and carbohydrates [34,37,39,50].

**Figure 2:**
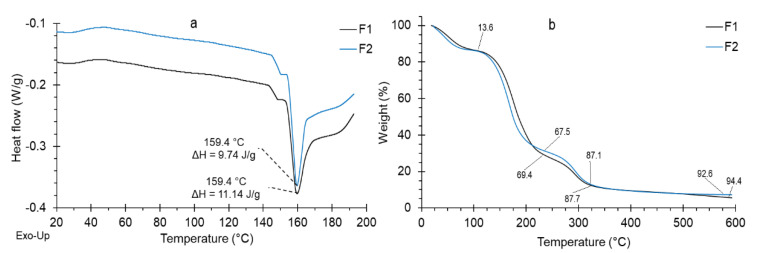
Thermal analysis of coatings, (**a**) DSC, (**b**) TGA.

The coatings must have good flexibility, and this is achieved with the addition of plasticizers such as glycerin. The low values of the glass transition temperatures show high flexibility, such as those found in the elaborated polymers (F1 and F2), with values around 29 °C (Figure 2a), with endothermic peaks and similar behavior. This is because they have the same glycerol content [49]. In the same way, it was observed that the gelatinization temperature was around 159.4 °C for both polymers, although, with a slightly higher gelatinization enthalpy for F1 (11.14 J/g). This is due to the higher content of pectin, which presents a greater number of branches in its structure than starch [34,37,49].

The TGA study showed that both F1 and F2 polymers retain moisture at around 110 °C, which means an average mass loss of 13.6% (Figure 2b). Likewise, it was observed that about 94.4% of the material used as a coating in the avocado is organic matter, so it can be considered a biodegradable material, and this behavior is similar for polymers of this nature [20,34,35,37].

### 3.2. Physicochemical Properties

#### 3.2.1. Acidity

Acidity is slightly higher for the coated and control of the Hass variety, while for the Fuerte variety, it reported 5.88, 4.71, and 4.94 for the control, F1, and F2, respectively (Table 3).

**Figure 3:**
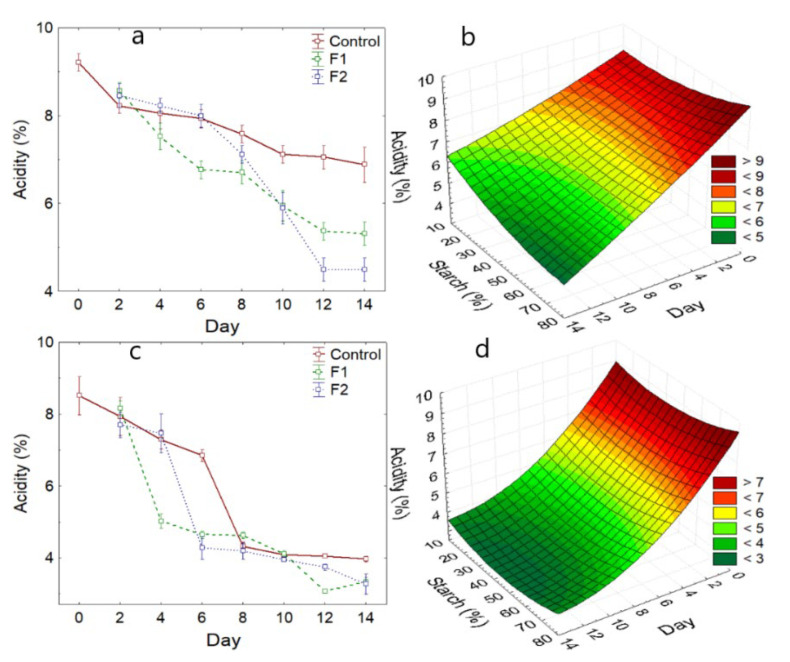
Acidity variation, (**a**) Hass variety, (**b**) with respect to the addition of starch—Hass variety, (**c**) Fuerte variety, (**d**) with respect to the addition of starch—Fuerte variety.

It was observed that the reported acidity for the Hass variety during storage was less decreased for the control sample (Figure 3a), while the F1 coating it was higher. In the same way, a considerable decrease was observed between days 6 to 12 for F1 and F2, respectively. For the Fuerte variety, a similar decrease occurred in the control and coated samples, although a strong drop occurred around day 6 (Figure 3c). This behavior is characteristic of fruits and vegetables due to the metabolic rate and coincides with the beginning of maturation and sugar accumulation [51].

The fact of acidity decrease is due to the activity of dehydrogenases, which behave as substrates for the synthesis of new products during maturation, generating higher sugar content and volatile substances, which give better sensory characteristics to coated avocados [2,52].

The addition of potato starch in the formulation of the coating allows the reduction of avocado acidity for both varieties (Figure 3b,d), due to the fact that it would achieve a greater impermeable capacity to gases and humidity.

#### 3.2.2. pH

**Figure 4:**
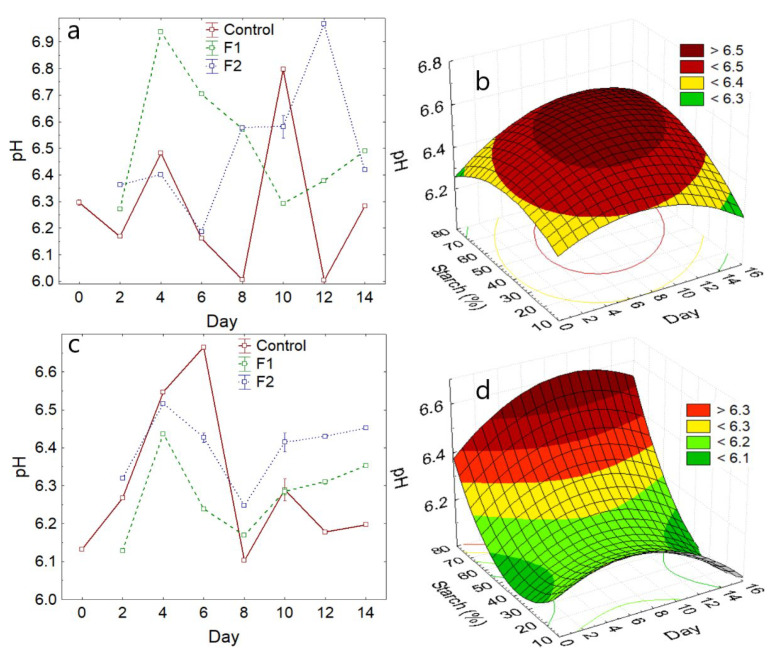
pH variation (**a**) Hass variety, (**b**) with respect to the addition of starch—Hass variety, (**c**) Fuerte variety, (**d**) with respect to the addition of starch—Fuerte variety.

It was observed that the pH values for the two varieties showed significant variations during the maturation time (*p*-value < 0.05) (Figure 4a,c). However, the mean value is slightly similar for the coated samples of both varieties (Table 4), indicating that the coating allows for the preservation of the pH of the fruits, this being a usual behavior for coated avocado [1,53].

This behavior is due to the fact that during the maturation stage, the pH increases due to the development of acidic substances, and once the maximum point of maturation is reached, these tend to decrease due to the fact that they are consumed in the metabolic processes. They also act as precursors of volatile substances in avocado, so the pH tends to neutrality [41,54].

On the other hand, increasing the addition of potato starch in the formulation of the coatings allows the pH of the avocado fruits of the two varieties to increase slightly (Figure 4b,d).

#### 3.2.3. Soluble Solids

**Figure 5:**
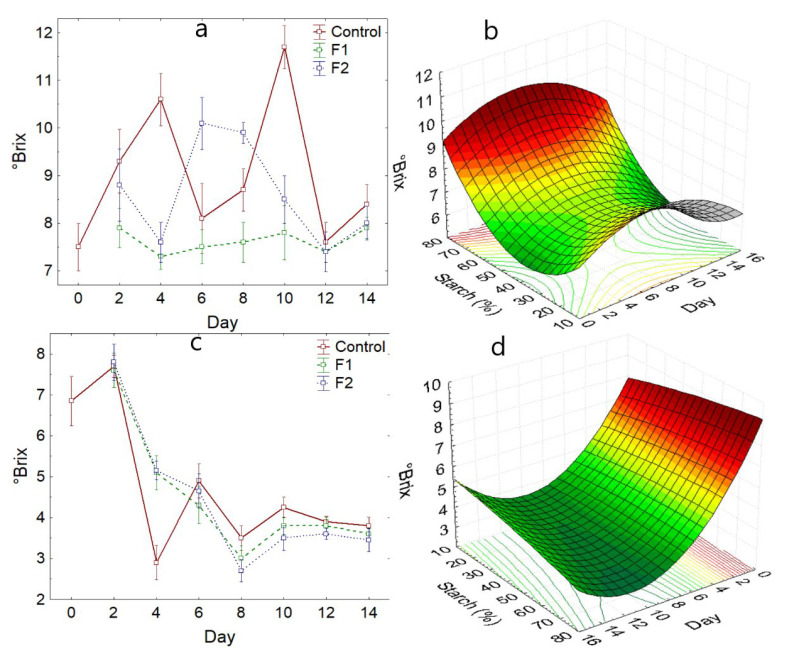
Soluble solids variation, (**a**) Hass variety, (**b**) with respect to the addition of starch—Hass variety, (**c**) Fuerte variety, (**d**) with respect to the addition of starch—Fuerte variety.

Regarding soluble solids, the Hass variety showed a higher value on days 4 to 10 (Figure 5a), and the control sample showed the opposite behavior (*p*-value < 0.05), with values around 7.0 °Brix (Table 5), which is characteristic of the Hass variety [1,38].

Regarding the Fuerte variety, the coated samples showed similar behavior during the storage time (*p*-value = 0.928), decreasing the soluble solids rapidly during the first 8 days of storage, and thereafter there is a slight variation reaching values around 4 °Brix (Figure 5c). In fact, the F2 coating shows lower values of soluble solids. This is due to the plasticizing effect of starch (Figure 5b,d), which prevents water loss, which favors maturation, increasing the concentration of sugars due to the phenomenon of respiration of this climacteric fruit [54,55].

The variation of soluble solids in avocado fruit would be involved in the enzymatic activity of alpha and beta-amylase, which hydrolyze starches to simple sugars [32]. The Hass variety would present a higher carbohydrate content because the soluble solids increase between days 4 and 10 (Figure 5a), while the Fuerte variety does not show this behavior. On the contrary, it decreases, confirming the higher matter fat content [53].

### 3.3. Evaluation of Physiological Properties

#### 3.3.1. Avocado Weight Loss

**Figure 6:**
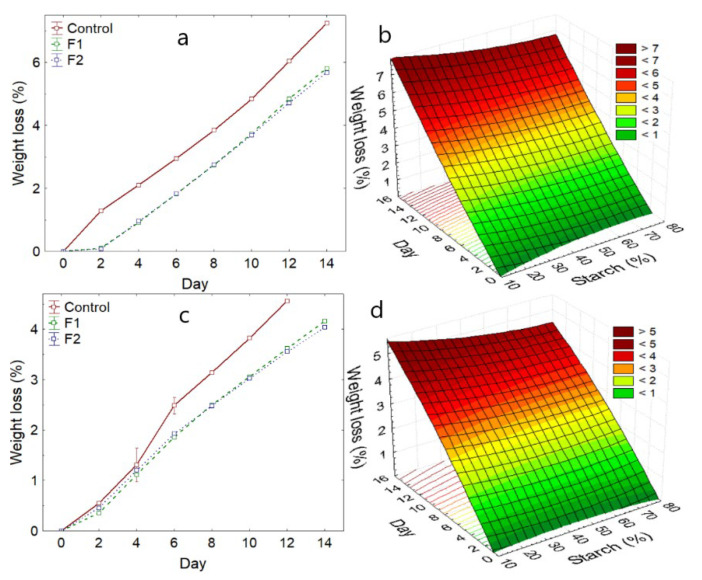
Variation of weight loss, (**a**) Hass variety, (**b**) with respect to the addition of starch—Hass variety, (**c**) Fuerte variety, (**d**) with respect to the addition of starch—Fuerte variety.

During storage, the weight of coated and control avocado fruits decreased significantly for both varieties (*p*-value < 0.05). The F2 formulation reported less loss, 5.19% and 3.79% for the Hass and Fuerte varieties, respectively (Table 6). Weight loss in both varieties manifests itself in an increasing way until day 14. Although the first two days are slower (Figure 6a,c).

Likewise, it was observed that the increase in starch in the formulations does not considerably influence the weight loss for both coated varieties (Figure 6b,d). This indicates that the starch offers good impermeability to water, offering a better barrier.

Weight loss during storage is mainly due to moisture loss, which is due to the water vapor pressure gradient between the fruit and the environment as well as cellular activity, and physiological processes in the fruit [15,18,35,38]. However, this loss can be lessened through the use of barriers such as films, which would prevent transpiration, and the exchange of gases in the fruit with the environment [18,52], although they could be affected by the environmental temperature and relative humidity [28,56].

Weight loss becomes an economic loss, which considerably affects producers, marketers, and exporters, reaching, in many cases, up to 60% of the total weight, and this is accompanied by the loss of sensory quality [23,57,58].

#### 3.3.2. Avocado Color

It was observed that the luminosity L*, for the control decreases considerably (*p*-value < 0.05), with a tendency to gray. In the coated samples, F1 and F2, there was less of a decrease reported at day 20 (Table 7). This would be due to the fact that the coating gives greater brightness to the fruits.

Chroma a* for the Fuerte variety increased with a similar trend for the control and the coated varieties (Table 7), acquiring a greater shade of dark green during storage time, while chroma b* decreased slightly for the control sample.

As for the Hass variety, L* decreases considerably for the control sample after 20 days of storage to values of 6.00, while for F1 and F2 it decreased from 29.80 to 9.10 and from 29.70 to 7.47 (Table 8). The chroma a* increases for the control sample from −15.77 to 1.63. In the same way, it occurs for the coated samples, that is, a considerable change from green to a red trend.

Negative a* values indicate a green tonality trend, and it is associated with the presence of chlorophyll b (3-methyl group). An increase in this parameter would indicate degradation due to enzymatic action, producing phytol and chlorophyllide [10,30,59,60]. This is manifested in both varieties, although with greater emphasis on the Hass variety (Table 7 and Table 8). This is a characteristic behavior for this coated and natural variety [26,28,35,36,52,61].

Chroma b* decreases considerably for both varieties, control and coated, and is associated with the presence of chlorophyll A with a blue tonality (3-formyl group), and at storage this tonality is attenuated [27,59,61]. At the same time, carotenoid pigments are synthesized and degraded, giving the fruit that dark coloration [18,62,63].

On the other hand, the color index (CI*) provides insight into the overall color trend [43]. Values between −20.0 to −2.0 indicate a trend from deep green to yellowish-green. During storage, the Fuerte variety showed a tendency to dark green, and it was more pronounced from day 10, being higher for the control sample, which indicates that the coatings allow for the maintenance of the intense green color, which is characteristic of the Fuerte variety. Regarding the Hass variety, it was observed that CI* increases from negative to positive values, from intense green to intense red (CI* > 20) (Table 8). This tonality is characteristic of the Hass variety in its state of maturity.

**Figure 7:**
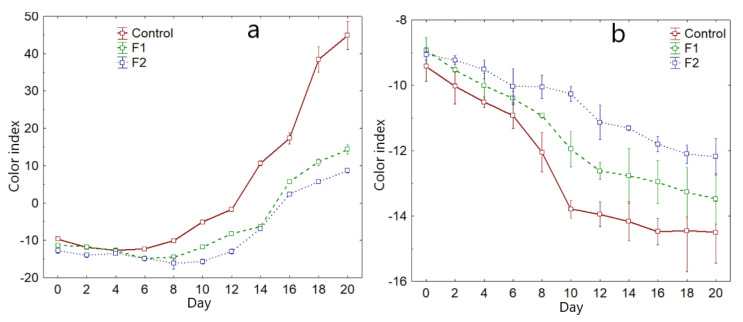
Color index variation, (**a**) Hass variety, (**b**) Fuerte variety.

In Figure 7a,b, a break in the curve is observed, which occurs approximately at 10 days for both varieties. This point would refer to the climacteric peak, where the fruit is completely mature [2,36]. However, coated fruits show less tendency to mature because the coating offers a barrier to the transfer of oxygen to the fruit, reducing metabolism and, consequently, CO_2_ production as well as reducing oxidation reactions at the cellular level. [18,23,56,61].

**Figure 8:**
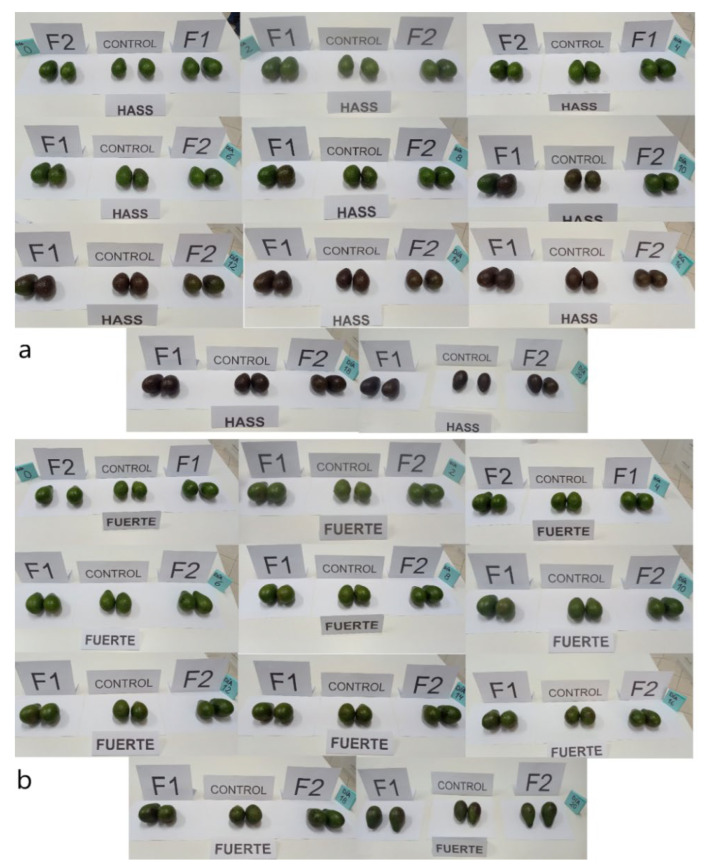
Color variation, (**a**) Hass variety, (**b**) Fuerte variety.

Regarding color variation (ΔE*), values greater than 3 are classified as very different compared to the initial sample. It was observed that the Hass and Fuerte varieties coated with F2 reported values of ΔE* < 3, for days 2 and 4 (Table 7 and Table 8), and thereafter it increased, although with less intensity for the samples coated with F2, which suggests that this coating presents better protection against fruit color deterioration. However, the fruits of the Hass variety acquire reddish tones while the Fuerte variety tends towards a dark green tone (Figure 8a,b).

#### 3.3.3. Fruit Firmness

Firmness showed a significant decrease (*p*-value < 0.05) during storage time (Table 9). As for the Fuerte variety, significant differences were observed between treatments (*p*-value < 0.05) at 20 days of storage, with the control sample reporting less firmness (0.30 kg_f_/cm^2^), and slightly higher for the samples coated with F2. (0.70 kg_f_/cm^2^). While, for the Hass variety, it was observed that the control sample presented firmness of 0.50 kg_f_/cm^2^, and for the samples coated with F2 it was 2.80 kg_f_/cm^2^ (*p*-value < 0.05).

**Figure 9:**
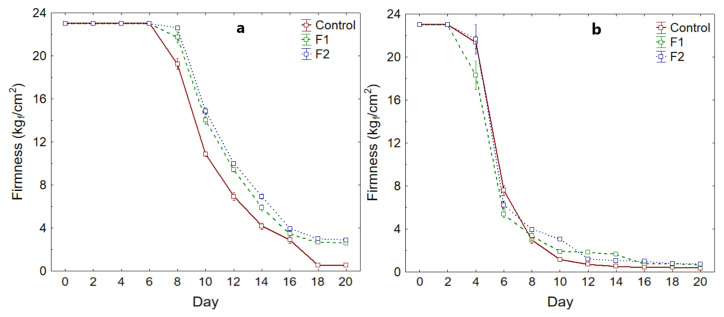
Firmness variation, (**a**) Hass variety, (**b**) Fuerte variety.

The beginning of the considerable decrease in firmness was around day 8, for both varieties (Figure 9a,b), which suggests that the samples reached full maturity, which is the climacteric peak [36,64]. From day 18 onwards for the Hass variety, and day 14 for the Fuerte variety, firmness stabilizes with minimum values being lower for the control samples.

This would be because the coatings allow for delayed degradation of protopectin into more soluble compounds, such as pectic acid and sugars, due to depolymerization of pectins by enzymatic action. Although, these are limited by the lack of oxygen due to the barrier action of the polymer [28,54,55,65,66]. In addition, another aspect is the low water loss of the coated fruits, which allows better firmness to be maintained [38,67], and this behavior is characteristic of samples coated with polymers [18,23,26,31,35,38,52,61,68].

## 4. Conclusions

Films made from native potato starch, nopal mucilage, and pectin showed high transparency and low *a_w_* values. Regarding the coated avocado of the Hass and Fuerte varieties, it was observed that the acidity and the total soluble solids decreased significantly (*p*-value < 0.05), although the pH did not vary considerably during the storage time. Related to weight loss and firmness, it was observed that they decreased to a lesser magnitude for both varieties. Regarding the luminosity L*, color index, and color variation, better attributes were observed for the coated samples of both varieties during 20 days of storage. The use of coatings formulated with potato starch and nopal mucilage allows for the maintenance of the physicochemical and physiological properties of avocado fruits of the Hass and Fuerte varieties for a longer time in storage.

## Figures and Tables

**Table 1:** Formulation of emulsions.

Formulation	PS% (at 3%)	NM% (at 2%)	G%	PC% (at 2%)
F1	60.0	4.0	4.0	32.0
F2	70.0	4.0	4.0	22.0

**Table 2:** Water activity and transparency (nm/mm) of the films.

Parameter	F1				F2				*p*-Value
	$\bar{x}$	±	SD	CV (%)	$\bar{x}$	±	SD	CV (%)	
*a_w_*	0.422	±	0.006	1.517	0.404	±	0.005	1.151	<0.05
Transmittance (%)	81.315	±	2.617	3.219	83.187	±	1.564	1.880	0.26
Transparency (nm/mm)	6.939	±	0.276	3.971	7.332	±	0.194	2.643	0.06

Where: $\bar{x}$ , arithmetic mean; SD, standard deviation; CV, coefficient of variability.

**Table 3:** Acidity (% citric acid) of control and coated avocados.

	Hass			Fuerte		
	Control	F1	F2	Control	F1	F2
Maximum	9.45	8.75	8.75	9.10	8.40	8.05
Minimum	6.65	5.08	4.20	3.88	3.03	3.05
$\bar{x}$ ****	7.76	6.60	6.67	5.88	4.71	4.94
DS	0.77	1.14	1.64	1.89	1.60	1.76
CV(%)	9.90	17.33	24.64	32.09	33.98	35.61
*p*-value *	<0.05	<0.05	<0.05	<0.05	<0.05	<0.05
*p*-value **	<0.05			<0.05		
*p*-value ***	<0.05			<0.05		

Where: $\bar{x}$ , arithmetic mean; SD, standard deviation; CV, coefficient of variability. * *p*-value per treatment, ** *p*-value per treatment comparison, *** *p*-value per day of maturation within treatments, **** For *n* = 3.

**Table 4:** pH of control and coated avocado.

	Hass			Fuerte		
	Control	F1	F2	Control	F1	F2
Maximum	6.80	6.94	6.97	6.67	6.44	6.52
Minimum	6.00	6.27	6.18	6.10	6.13	6.24
$\bar{x}$ ****	6.27	6.52	6.50	6.30	6.27	6.40
DS	0.25	0.23	0.23	0.19	0.10	0.08
CV(%)	3.98	3.45	3.57	3.06	1.59	1.32
*p*-value *	<0.05	<0.05	<0.05	<0.05	<0.05	<0.05
*p*-value **	<0.05			<0.05		
*p*-value ***	<0.05			<0.05		

Where: $\bar{x}$ , arithmetic mean; SD, standard deviation; CV, coefficient of variability. * *p*-value per treatment, ** *p*-value per treatment comparison, *** *p*-value per day of maturation within treatments, **** For *n* = 3.

**Table 5:** Soluble solids (°Brix) of control and coated avocado.

	Hass			Fuerte		
	Control	F1	F2	Control	F1	F2
Maximum	12.00	8.50	11.00	8.00	8.00	8.50
Minimum	7.00	7.00	7.00	2.50	2.75	2.50
$\bar{x}$ ****	8.99	7.63	8.61	4.73	4.46	4.41
DS	1.49	0.43	1.09	1.63	1.47	1.63
CV (%)	16.55	5.58	12.68	34.56	32.92	36.89
*p*-value *	<0.05	0.1	<0.05	<0.05	<0.05	<0.05
*p*-value **	<0.05			<0.05		
*p*-value ***	<0.05			0.928		

Where: $\bar{x}$ , arithmetic mean; SD, standard deviation; CV, coefficient of variability. * *p*-value per treatment, ** *p*-value per treatment comparison, *** *p*-value per day of maturation within treatments, **** For *n* = 3.

**Table 6:** Weight loss (%) of control and coated avocado.

	Hass			Fuerte		
	Control	F1	F2	Control	F1	F2
Maximum	7.25	5.81	5.67	5.10	4.16	4.04
Minimum	6.03	4.85	4.71	4.56	3.62	3.55
$\bar{x}$ ****	6.64	5.33	5.19	4.83	3.89	3.79
DS	0.66	0.53	0.53	0.30	0.30	0.26
CV (%)	9.98	9.90	10.12	6.18	7.65	6.97
*p*-value *	<0.05	<0.05	<0.05	<0.05	<0.05	<0.05
*p*-value **	0.150			0.817		
*p*-value ***	<0.05			<0.05		

Where: $\bar{x}$ , arithmetic mean; SD, standard deviation; CV, coefficient of variability. * *p*-value per treatment, ** *p*-value per treatment comparison, *** *p*-value per day of maturation within treatments, **** For *n* = 3.

**Table 7:** Color of Fuerte variety avocado.

Day	L*				a*				b*				CI*					ΔE *				Referential Color
	$\bar{x}$	±	SD	CV	$\bar{x}$	±	SD	CV	$\bar{x}$	±	SD	CV	$\bar{x}$	±	SD	CV	*	$\bar{x}$	±	SD	CV	
	Control																					
0	32.90	±	0.78	2.37	−17.30	±	1.15	6.67	55.77	±	0.21	0.37	−9.42	±	0.45	4.79	a					
2	32.73	±	0.32	0.98	−18.73	±	0.70	3.75	57.10	±	0.56	0.98	−10.03	±	0.54	5.40	a	2.34	±	0.52	22.25	
4	24.97	±	0.21	0.83	−16.23	±	0.15	0.94	61.83	±	0.31	0.49	−10.52	±	0.17	1.62	a,b	10.09	±	0.98	9.68	
6	22.23	±	0.38	1.70	−13.83	±	0.25	1.82	56.97	±	0.29	0.51	−10.93	±	0.40	3.65	a,b	11.33	±	0.80	7.03	
8	21.07	±	0.25	1.19	−13.67	±	0.51	3.75	53.87	±	0.47	0.88	−12.05	±	0.61	5.06	b,c	12.57	±	1.15	9.18	
10	19.80	±	0.70	3.54	−13.93	±	0.25	1.81	51.07	±	0.35	0.69	−13.79	±	0.27	1.95	c,d	14.35	±	0.65	4.55	
12	17.83	±	0.49	2.77	−12.60	±	0.44	3.46	50.67	±	1.06	2.09	−13.95	±	0.38	2.71	d	16.67	±	1.22	7.29	
14	17.63	±	0.50	2.85	−12.93	±	0.15	1.18	51.83	±	0.50	0.97	−14.16	±	0.60	4.22	d	16.39	±	1.19	7.25	
16	15.37	±	0.31	1.99	−11.59	±	0.50	4.28	52.07	±	0.40	0.78	−14.48	±	0.40	2.76	d	18.81	±	0.81	4.31	
18	12.93	±	0.38	2.93	−9.93	±	0.51	5.17	53.23	±	0.42	0.78	−14.45	±	1.25	8.63	d	21.45	±	1.17	5.47	
20	10.87	±	0.21	1.92	−8.40	±	0.36	4.29	53.37	±	0.67	1.25	−14.50	±	0.94	6.51	d	23.90	±	0.97	4.08	
	**F1**																					
0	36.17	±	0.75	2.08	−17.77	±	1.38	7.77	55.00	±	0.78	1.42	−8.92	±	0.40	4.44	a					
2	36.27	±	0.42	1.15	−17.87	±	0.40	2.26	51.70	±	0.40	0.77	−9.53	±	0.08	0.82	a,b	3.73	±	0.62	16.71	
4	34.83	±	0.31	0.88	−17.77	±	0.40	2.27	50.97	±	0.32	0.63	−10.01	±	0.37	3.73	a,b	4.46	±	1.03	23.16	
6	33.77	±	0.31	0.90	−17.80	±	0.26	1.49	50.70	±	0.36	0.71	−10.40	±	0.21	2.01	a,b	5.13	±	0.80	15.52	
8	33.13	±	0.59	1.77	−15.83	±	0.25	1.59	43.73	±	0.45	1.03	−10.93	±	0.08	0.69	b.c	11.87	±	1.08	9.09	
10	30.13	±	0.60	2.00	−14.67	±	0.46	3.15	40.77	±	0.46	1.13	−11.95	±	0.54	4.55	c,d	15.80	±	1.83	11.59	
12	27.27	±	0.45	1.65	−13.13	±	0.21	1.59	38.17	±	0.40	1.06	−12.62	±	0.27	2.11	d	19.62	±	1.06	5.40	
14	25.27	±	0.57	2.25	−12.00	±	0.44	3.63	37.23	±	0.64	1.73	−12.77	±	0.83	6.53	d	21.68	±	0.90	4.13	
16	22.23	±	0.45	2.03	−10.67	±	0.51	4.81	37.03	±	0.67	1.80	−12.96	±	0.65	5.02	d	23.85	±	1.63	6.85	
18	18.13	±	0.55	3.04	−8.90	±	0.26	2.97	37.03	±	0.21	0.56	−13.27	±	0.76	5.71	d	26.98	±	1.38	5.11	
20	18.00	±	0.26	1.47	−9.00	±	0.36	4.01	37.13	±	0.35	0.95	−13.47	±	0.78	5.78	d	26.97	±	1.49	5.53	
	**F2**																					
0	36.80	±	0.40	1.09	−19.00	±	0.26	1.39	57.03	±	0.67	1.17	−9.05	±	0.19	2.07	a					
2	36.20	±	0.44	1.20	−18.97	±	0.21	1.10	56.73	±	0.21	0.37	−9.24	±	0.15	1.58	a	1.08	±	0.21	19.81	
4	34.23	±	0.46	1.35	−18.80	±	0.53	2.81	57.73	±	0.60	1.04	−9.51	±	0.29	3.05	a,b	2.84	±	0.89	31.39	
6	33.70	±	0.26	0.79	−15.77	±	0.29	1.83	46.70	±	1.73	3.71	−10.03	±	0.55	5.44	a,b	11.32	±	1.58	13.93	
8	33.20	±	0.36	1.09	−15.47	±	0.32	2.08	46.37	±	0.59	1.26	−10.05	±	0.36	3.54	a,b	11.80	±	0.30	2.56	
10	31.60	±	0.44	1.38	−14.87	±	0.21	1.40	45.83	±	0.55	1.20	−10.27	±	0.23	2.24	b,c	13.03	±	0.37	2.82	
12	29.30	±	0.66	2.24	−14.23	±	0.51	3.61	43.67	±	0.90	2.07	−11.13	±	0.52	4.69	c,d	16.07	±	1.28	7.99	
14	26.60	±	0.40	1.50	−13.70	±	0.35	2.53	45.53	±	1.05	2.31	−11.31	±	0.07	0.62	d,e	16.28	±	1.19	7.30	
16	20.93	±	0.32	1.54	−10.90	±	0.26	2.43	44.13	±	1.27	2.88	−11.80	±	0.24	2.00	d,e	22.00	±	1.41	6.39	
18	18.77	±	0.31	1.63	−9.83	±	0.06	0.59	43.30	±	0.46	1.06	−12.10	±	0.28	2.29	d,e	24.45	±	0.46	1.87	
20	18.17	±	0.42	2.29	−9.67	±	0.42	4.31	43.70	±	0.61	1.39	−12.18	±	0.56	4.58	e	24.76	±	0.47	1.91	

Where: $\bar{x}$ , arithmetic mean; SD, standard deviation; CV, coefficient of variability. * Different letters indicate significant difference, evaluated through Tukey’s test at 5% significance.

**Table 8:** Color of Hass variety avocado.

Day	L*				a*				b*				CI*					ΔE*				Referential Color
	$\bar{x}$	±	SD	CV	$\bar{x}$	±	SD	CV	$\bar{x}$	±	SD	CV	$\bar{x}$	±	SD	CV	*	$\bar{x}$	±	SD	CV	
	Control																					
0	29.73	±	0.51	1.73	−15.77	±	0.45	2.86	55.10	±	0.61	1.10	−9.62	±	0.20	2.11	f,g					
2	26.60	±	0.46	1.72	−15.80	±	0.20	1.27	50.07	±	0.15	0.31	−11.86	±	0.09	0.77	g	5.99	±	0.48	8.01	
4	22.83	±	0.21	0.91	−13.50	±	0.61	4.51	46.60	±	0.30	0.64	−12.69	±	0.72	5.64	g	11.21	±	0.76	6.79	
6	19.83	±	0.55	2.78	−10.63	±	0.55	5.18	43.53	±	0.45	1.04	−12.31	±	0.42	3.41	g	16.10	±	1.02	6.35	
8	19.73	±	0.80	4.06	−8.77	±	0.50	5.74	43.77	±	0.35	0.80	−10.15	±	0.27	2.62	g	16.70	±	1.20	7.21	
10	18.70	±	0.50	2.67	−4.07	±	0.25	6.19	42.33	±	0.47	1.12	−5.13	±	0.13	2.47	e,f	20.54	±	0.78	3.77	
12	14.17	±	0.35	2.48	−0.53	±	0.06	10.83	22.17	±	0.71	3.20	−1.70	±	0.10	6.16	e	39.49	±	0.61	1.54	
14	12.10	±	0.20	1.65	1.47	±	0.12	7.87	11.40	±	0.46	4.02	10.63	±	0.67	6.31	d	50.18	±	0.66	1.32	
16	6.93	±	0.35	5.07	1.33	±	0.12	8.66	11.10	±	0.17	1.56	17.34	±	1.50	8.67	c	52.43	±	0.64	1.22	
18	5.80	±	0.40	6.90	1.47	±	0.12	7.87	6.60	±	0.36	5.46	38.44	±	3.42	8.89	b	56.77	±	0.75	1.32	
20	6.00	±	0.17	2.89	1.63	±	0.15	9.35	6.07	±	0.25	4.15	44.89	±	3.80	8.47	a	57.19	±	0.53	0.93	
	**F1**																					
0	29.80	±	0.52	1.74	−18.63	±	0.40	2.17	55.20	±	0.56	1.01	−11.33	±	0.48	4.22	f					
2	26.50	±	0.26	1.00	−17.27	±	0.45	2.61	55.47	±	0.25	0.45	−11.75	±	0.40	3.38	f	3.70	±	0.27	7.35	
4	25.33	±	0.06	0.23	−16.67	±	0.55	3.30	51.23	±	0.47	0.92	−12.84	±	0.56	4.34	f,g	6.32	±	0.39	6.17	
6	21.63	±	0.64	2.97	−14.10	±	0.40	2.84	43.83	±	0.42	0.95	−14.88	±	0.70	4.69	h	14.72	±	0.49	3.36	
8	20.43	±	1.07	5.23	−11.90	±	0.40	3.36	40.07	±	0.55	1.37	−14.55	±	0.36	2.46	g,h	19.07	±	0.29	1.50	
10	17.80	±	0.30	1.69	−8.20	±	0.26	3.23	39.00	±	0.46	1.18	−11.81	±	0.23	1.97	f	22.71	±	0.69	3.03	
12	16.70	±	0.46	2.74	−3.97	±	0.15	3.85	28.87	±	0.81	2.82	−8.24	±	0.42	5.04	e	32.87	±	0.78	2.37	
14	15.00	±	0.36	2.40	−2.07	±	0.15	7.39	21.83	±	0.55	2.52	−6.30	±	0.25	3.99	d	40.09	±	0.54	1.34	
16	11.77	±	0.67	5.66	0.93	±	0.06	6.19	13.80	±	0.56	4.03	5.75	±	0.17	2.96	c	49.22	±	0.83	1.68	
18	10.23	±	0.40	3.95	1.17	±	0.12	9.90	10.37	±	0.15	1.47	11.00	±	0.94	8.57	b	52.77	±	0.58	1.09	
20	9.10	±	0.20	2.20	1.20	±	0.10	8.33	9.20	±	0.26	2.88	14.35	±	1.29	8.99	a	54.20	±	0.46	0.84	
	**F2**																					
0	29.70	±	0.26	0.89	−18.17	±	0.68	3.75	47.90	±	0.44	0.91	−12.78	±	0.67	5.21	e		±			
2	27.80	±	0.26	0.95	−18.40	±	0.69	3.77	47.30	±	0.46	0.97	−13.99	±	0.53	3.80	e,f,g	2.31	±	0.16	7.06	
4	25.67	±	0.21	0.81	−16.20	±	0.46	2.83	46.80	±	0.82	1.75	−13.49	±	0.24	1.80	e,f	4.71	±	0.23	4.90	
6	21.13	±	0.21	0.99	−14.73	±	0.71	4.82	46.97	±	0.61	1.30	−14.84	±	0.48	3.22	f,g,h	9.31	±	0.42	4.54	
8	20.17	±	0.93	4.61	−13.63	±	0.50	3.69	42.00	±	0.46	1.09	−16.15	±	1.48	9.18	h	12.10	±	0.60	4.95	
10	18.23	±	0.42	2.28	−11.97	±	0.42	3.48	41.93	±	0.49	1.18	−15.66	±	0.64	4.12	g,h	14.36	±	0.46	3.22	
12	17.37	±	0.51	2.95	−8.13	±	0.47	5.81	36.10	±	0.53	1.47	−12.98	±	0.71	5.45	e,f	19.81	±	0.44	2.20	
14	13.33	±	0.15	1.15	−2.93	±	0.15	5.21	32.13	±	0.25	0.78	−6.85	±	0.45	6.62	d	27.37	±	0.31	1.13	
16	9.80	±	0.60	6.12	0.63	±	0.06	9.12	27.47	±	0.65	2.37	2.35	±	0.17	7.10	c	34.17	±	0.18	0.52	
18	7.87	±	0.31	3.88	0.93	±	0.06	6.19	20.77	±	0.31	1.47	5.71	±	0.12	2.06	b	39.72	±	0.40	1.00	
20	7.47	±	0.15	2.05	1.33	±	0.12	8.66	20.60	±	0.66	3.18	8.67	±	0.63	7.31	a	40.26	±	0.54	1.35	

Where: $\bar{x}$ , arithmetic mean; SD, standard deviation; CV, coefficient of variability. * Different letters indicate significant difference, evaluated through Tukey’s test at 5% significance.

**Table 9:** Firmness (kg_f_/cm^2^) of control and coated avocados.

	Hass			Fuerte		
	Control	F1	F2	Control	F1	F2
Maximum	23.00	23.00	23.00	23.00	23.00	23.00
Minimum	0.50	2.50	2.80	0.30	0.60	0.70
$\bar{x}$ ****	12.47	13.80	14.20	7.42	7.32	7.78
SD	9.53	8.88	8.72	9.58	8.96	9.34
CV (%)	76.41	64.34	61.39	129.22	122.28	119.98
*p*-value *	<0.05	<0.05	<0.05	<0.05	<0.05	<0.05
*p*-value **	<0.05			<0.05		
*p*-value ***	<0.05			<0.05		

Where: $\bar{x}$ , arithmetic mean; SD, standard deviation; CV, coefficient of variability. * *p*-value per treatment, ** *p*-value per treatment comparison, *** *p*-value per day of maturation within treatments, **** For *n* = 3.

## Data Availability

The data presented in this study are available in this same article.

## References

[1] Benítez J., Sánchez A., Bolaños C., Bernal L., Ochoa-Martínez C., Vélez C., Sandoval A. (2021). Physicochemical changes of avocado Hass during cold storage and accelerated ripening. Biotecnol. Sect. Agropecu. Agroind..

[2] Park Y.S., Jung S.T., Gorinstein S. (2006). Ethylene treatment of ‘Hayward’ kiwifruits (*Actinidia deliciosa*) during ripening and its influence on ethylene biosynthesis and antioxidant activity. Sci. Hortic..

[3] Villa-Rodriguez J.A., Yahia E.M., González-León A., Ifie I., Robles-Zepeda R.E., Domínguez-Avila J.A., González-Aguilar G.A. (2020). Ripening of “Hass” avocado mesocarp alters its phytochemical profile and the in vitro cytotoxic activity of its methanolic extracts. South Afr. J. Bot..

[4] Escobar J., Rodríguez P., Cortés M., Correa G. (2019). Influence of dry matter as a harvest index and cold storage time on cv. Hass avocado quality produced in high tropic region. Inf. Tecnol..

[5] Araújo R.G., Rodriguez-Jasso R.M., Ruiz H., Pintado M.M.E., Aguilar C.N. (2018). Avocado by-products: Nutritional and functional properties. Trends Food Sci. Technol..

[6] Ferreyra R., Sellés G., Saavedra J., Ortiz J., Zúñiga C., Troncoso C., Rivera S., González-Agüero M., Defilippi B. (2016). Identification of pre-harvest factors that affect fatty acid profiles of avocado fruit (*Persea americana* Mill) cv. ‘Hass’ at harvest. S. Afr. J. Bot..

[7] Donetti M., Terry L.A. (2014). Biochemical markers defining growing area and ripening stage of imported avocado fruit cv. Hass. J. Food Compos. Anal..

[8] Dreher M.L., Davenport A.J. (2013). Hass avocado composition and potential health effects. Crit. Rev. Food Sci. Nutr..

[9] Pedreschi R., Uarrota V., Fuentealba C., Alvaro J.E., Olmedo P., Defilippi B.G., Meneses C., Campos-Vargas R. (2019). Primary Metabolism in Avocado Fruit. Front. Plant Sci..

[10] Xoca-Orozco L.A., Aguilera-Aguirre S., López-García U.M., Gutiérrez-Martínez P., Chacón-López M.A. (2019). Effect of chitosan on the in vitro control of *Colletotrichum* sp., and its influence on post-harvest quality in Hass avocado fruits. Rev. Bio Cienc..

[11] Tochihuitl-Martiñón A., Chávez-Franco S.H., Saucedo-Veloz C., Suarez-Espinosa J., Guerra-Ramírez D. (2018). Extracts of *Persea americana* Mill. that delay ripeningin avocado fruits. Rev. Mex. Cienc. Agríc..

[12] Arpaia M.L., Collin S., Sievert J., Obenland D. (2018). ‘Hass’ avocado quality as influenced by temperature and ethylene prior to and during final ripening. Postharvest Biol. Technol..

[13] Wang Q., Liu W., Tian B., Li D., Liu C., Jiang B., Feng Z. (2020). Preparation and Characterization of Coating Based on Protein Nanofibers and Polyphenol and Application for Salted Duck Egg Yolks. Foods.

[14] Dhall R. (2013). Advances in edible coatings for fresh fruits and vegetables: A Review. Crit Rev. Food Sci Nutr..

[15] Iñiguez-Moreno M., Ragazzo-Sánchez J.A., Barros-Castillo J.C., Sandoval-Contreras T., Calderón-Santoyo M. (2020). Sodium alginate coatings added with Meyerozyma caribbica: Postharvest biocontrol of Colletotrichum gloeosporioides in avocado (*Persea americana* Mill. cv. Hass). Postharvest Biol. Technol..

[16] Arpaia M., Requejo-Jackman C., Wollf A., Thompson J., Slaughter D., Tokar V. (2006). Avocado Postharvest quality. Proceedings of the California Avocado Research Symposium.

[17] Perkins M.L., Joyce D.C., Coates L.M. (2019). Possible contribution of impact injury at harvest to anthracnose expression in ripening avocado: A review. Sci. Hortic..

[18] Careli-Gondim I., Mesquita T.C., Boas E.V.D.B.V., Caliari M., Júnior M.S.S. (2020). The effect of active coating and refrigerated storage on the quality of avocado cultivar, Quintal. J. Food Sci. Technol..

[19] Landero-Valenzuela N., Lara-Viveros F.M., Andrade-Hoyos P., Aguilar-Pérez L.A., Aguado Rodríguez G.J. (2016). Alternatives for the control of *Colletotrichum* spp.. Rev. Mex. Cienc. Agríc..

[20] Ghaderi M., Mousavi M., Yousefi H., Labbafi M. (2014). All-cellulose nanocomposite film made from bagasse cellulose nanofibers for food packaging application. Carbohydr. Polym..

[21] Fuentealba C., Vidal J., Zulueta C., Ponce E., Uarrota V., Defilippi B.G., Pedreschi R. (2022). Controlled Atmosphere Storage Alleviates Hass Avocado Black Spot Disorder. Horticulturae.

[22] Maftoonazad N., Ramaswamy H.S., Moalemiyan M., Kushalappa A.C. (2007). Effect of pectin-based edible emulsion coating on changes in quality of avocado exposed to Lasiodiplodia theobromae infection. Carbohydr. Polym..

[23] Kubheka S.F., Tesfay S.Z., Mditshwa A., Magwaza L.S. (2020). Evaluating the efficacy of edible coatings incorporated with moringa leaf extract on postharvest of “maluma” avocado fruit quality and its biofungicidal effect. HortScience.

[24] Maran J.P., Sivakumar V., Sridhar R., Immanuel V.P. (2013). Development of model for mechanical properties of tapioca starch based edible films. Ind. Crops Prod..

[25] Quintão S.D.P., Oshiro A.M., Mugnol D. (2012). Postharvest conservation of guavira (*Campomanesia adamantium* Camb.) under different coating and temperatures of storage. Rev. Bras. Frutic. Jaboticabal.

[26] Scalon S.D., Oshiro A.M., Dresch D.M. (2015). Coating effect of Modified cassava starch in Hass avocado. Prod. Limpia.

[27] Aguilar-Méndez M.A., Martín-Martínez E.S., Tomás S.A., Cruz-Orea A., Jaime-Fonseca M.R. (2008). Gelatine–starch films: Physicochemical properties and their application in extending the post-harvest shelf life of avocado (*Persea americana*). J. Sci. Food Agric..

[28] Maftoonazad N., Ramaswamy H. (2005). Postharvest shelf-life extension of avocados using methyl cellulose-based coating. LWT-Food Sci. Technol..

[29] Granada D., López-Lujan L., Ramírez-Restrepo S., Morales J., Peláez-Jaramillo C., Andrade G., Bedoya-Pérez J.C. (2020). Bacterial extracts and bioformulates as a promising control of fruit body rot and root rot in avocado cv. Hass. J. Integr. Agric..

[30] Olivares D., Alvarez E., Véliz D., García-Rojas M., Díaz C., Defilippi B.G. (2020). Effects of 1-Methylcyclopropene and Controlled Atmosphere on Ethylene Synthesis and Quality Attributes of Avocado cvs. Edranol and Fuerte. J. Food Qual..

[31] Tesfay S.Z., Magwaza L.S., Mbili N., Mditshwa A. (2017). Carboxyl methylcellulose (CMC) containing moringa plant extracts as new postharvest organic edible coating for Avocado (*Persea americana* Mill.) fruit. Sci. Hortic..

[32] Kader A. (2004). Increasing food availability by reducing potharvest losses of fresh produce. Int. Postharvest Symp..

[33] Osondu H.A.A., Akinola S.A., Shoko T., Pillai S.K., Sivakumar D. (2022). Coating properties, resistance response, molecular mechanisms and anthracnose decay reduction in green skin avocado fruit (‘Fuerte’) coated with chitosan hydrochloride loaded with functional compounds. Postharvest Biol. Technol..

[34] Choque-Quispe D., Froehner S., Ligarda-Samanez C.A., Ramos-Pacheco B.S., Palomino-Rincón H., Choque-Quispe Y., Solano-Reynoso A.M., Taipe-Pardo F., Zamalloa-Puma L.M., Calla-Florez M. (2021). Preparation and Chemical and Physical Characteristics of an Edible Film Based on Native Potato Starch and Nopal Mucilage. Polymers.

[35] Iñiguez-Moreno M., Ragazzo-Sánchez J.A., Barros-Castillo J.C., Solís-Pacheco J.R., Calderón-Santoyo M. (2021). Characterization of sodium alginate coatings with Meyerozyma caribbica and impact on quality properties of avocado fruit. LWT-Food Sci. Technol..

[36] Garcia F., Davidov-Pardo G. (2021). Recent advances in the use of edible coatings for preservation of avocados: A review. J. Food Sci..

[37] Valdés A., Martínez C., Garrigos M.C., Jimenez A. (2021). Multilayer Films Based on Poly(lactic acid)/Gelatin Supplemented with Cellulose Nanocrystals and Antioxidant Extract from Almond Shell By-Product and Its Application on Hass Avocado Preservation. Polymers.

[38] Aguirre-Joya J.A., Ventura-Sobrevilla J., Martínez-Vazquez G., Ruelas-Chacón X., Rojas R., Rodríguez-Herrera R., Aguilar C.N. (2017). Effects of a natural bioactive coating on the quality and shelf life prolongation at different storage conditions of avocado (*Persea americana* Mill.) cv. Hass. Food Packag. Shelf Life.

[39] Choque-Quispe D., Ramos-Pacheco B.S., Ligarda-Samanez C.A., Barboza-Palomino G.I., Kari-Ferro A., Taipe-Pardo F., Choque-Quispe Y. (2022). Heavy metal removal by biopolymers-based formulations with native potato starch/nopal mucilage. Rev. Fac. Ing. Univ. Antioq..

[40] AOAC (2016). Official Methods of Analysis.

[41] Márquez Cardozo C.J., Yepes Betancur D.P., Sanchez Giraldo L., Osorio Saraz J.A. (2014). Changes physical-chemical of avocado (*Persea americana* Mill. cv. “Hass”) in postharvest for two municipalities of Antioquia. Rev. Temas Agrar..

[42] Choque-Quispe D., Ramos-Pacheco B.S., Solano-Reynoso A.M., Ligarda-Samanez C.A., Choque-Quispe Y., Peralta-Guevara D.E., Quispe-Quispe Y. (2021). Drying and color in punamuña leaves (*Satureja boliviana*). Dyna.

[43] Hadimani L., Mittal N. (2019). Development of a computer vision system to estimate the colour indices of Kinnow mandarins. J. Food Sci Technol..

[44] Adekunte A., Tiwari B., Cullen P., Scannell A., O’Donnell C. (2010). Effect of sonication on colour, ascorbic acid and yeast inactivation in tomato juice. Food Chem..

[45] Cheng M., Wang J., Zhang R., Kong R., Lu W., Wang X. (2019). Characterization and application of the microencapsulated carvacrol/sodium alginate films as food packaging materials. Int. J. Biol. Macromol..

[46] Schmid M., Sängerlaub S., Wege L., Stäbler A. (2014). Properties of transglutaminase crosslinked whey protein isolate coatings and cast films. Packag. Technol. Sci..

[47] Guldas M., Bayizit A.A., Yilsay T.O., Yilmaz L. (2010). Effects of edible film coatings on shelf-life of mustafakemalpasa sweet, a cheese based dessert. J. Food Sci. Technol..

[48] Guillard V., Broyart B., Bonazzi C., Guilbert S., Gontard N. (2004). Effect of Temperature on Moisture Barrier Efficiency of Monoglyceride Edible Films in Cereal-Based Composite Foods. Cereal Chem..

[49] Kibar E.A.A., Us F. (2013). Thermal, mechanical and water adsorption properties of corn starch–carboxymethylcellulose/methylcellulose biodegradable films. J. Food Eng..

[50] Muscat D., Adhikari B., Adhikari R., Chaudhary D.S. (2012). Comparative study of film forming behaviour of low and high amylose starches using glycerol and xylitol as plasticizers. J. Food Eng..

[51] Botía-Niño Y.C., Almanza-Merchán P., Balaguera-López H.E. (2008). Temperature effect on the complementary madurity in banana passion fruit (Passiflora mollissima Bailey). Rev. UDCA Actual. Divulg. Científica.

[52] Cenobio-Galindo A.D.J., Ocampo-López J., Reyes-Munguía A., Carrillo-Inungaray M.L., Cawood M., Medina-Pérez G., Fernández-Luqueño F., Campos-Montiel R.G. (2019). Influence of bioactive compounds incorporated in a nanoemulsion as coating on avocado fruits (*Persea americana*) during postharvest storage: Antioxidant activity, physicochemical changes and structural evaluation. Antioxidants.

[53] Astudillo-Ordóñez C.E., Rodríguez P. (2018). Physicochemical parameters of avocado *Persea americana* Mill. cv. Hass (*Lauraceae*) grown in Antioquia (Colombia) for export. Cienc. Tecnol. Agropecu..

[54] Buelvas-Salgado G., Patiño-Gómez J., Cano-Salazar J. (2012). Evaluation of the oil extraction from hasavocado (*Persea americana* Mill) by the use of an enzymatic treatment. Rev. Lasallista Investig..

[55] Saucedo-Pompa S., Rojas-Molina R., Aguilera-Carbó A.F., Saenz-Galindo A., de La Garza H., Jasso-Cantú D., Aguilar C.N. (2009). Edible film based on candelilla wax to improve the shelf life and quality of avocado. Food Res. Int..

[56] Cazón P., Velazquez G., Ramírez J.A., Vázquez M. (2017). Polysaccharide-based films and coatings for food packaging: A review. Food Hydrocoll..

[57] Jafarzadeh S., Nafchi A.M., Salehabadi A., Oladzad-Abbasabadi N., Jafari S.M. (2021). Application of bio-nanocomposite films and edible coatings for extending the shelf life of fresh fruits and vegetables. Adv. Colloid Interface Sci..

[58] Opara O., Mditshwa A. (2013). A review on the role of packaging in securing food system: Adding value to food products and reducing losses and waste. Afr. J. Agric. Res..

[59] Vincent C., Mesa T., Munne-Bosch S. (2020). Identification of a new variety of avocados (*Persea americana* Mill. CV. Bacon) with high vitamin E and impact of cold storage on tocochromanols composition. Antioxidants.

[60] Yang X., Zhang Z., Joyce D., Huang X., Xu L., Pang X. (2009). Characterization of chlorophyll degradation in banana and plantain during ripening at high temperature. Food Chem..

[61] Garcia F., Lin W.J., Mellano V., Davidov-Pardo G. (2022). Effect of biopolymer coatings made of zein nanoparticles and ε-polylysine as postharvest treatments on the shelf-life of avocados (*Persea americana* Mill. Cv. Hass). J. Agric. Food Res..

[62] Villa-Rodríguez J.A., Molina-Corral F.J., Ayala-Zavala J.F., Olivas G.I., González-Aguilar G.A. (2011). Effect of maturity stage on the content of fatty acids and antioxidant activity of ‘Hass’ avocado. Food Res. Int..

[63] Islam S., Matsui T., Yoshida Y. (1996). Effect of carbon dioxide enrichment on physico-chemical and enzymatic changes in tomato fruits at various stages of maturity. Sci. Hortic..

[64] De La Vega J., Cañarejo M., Pinto N. (2017). Avances en tecnología de atmósferas controladas y sus aplicaciones en la industria. Una revisión. Cienc. Agropecu. Ambient..

[65] Dhalsamant K., Mangaraj S., Bal L.M. (2017). Modified atmosphere packaging for mango and tomato: An appraisal to improve shelf life. J. Packag. Technol. Res..

[66] Wu B., Quilot B., Génard M., Kervella J., Li S. (2005). Changes in sugar and organic acid concentrations during fruit maturation in peaches, *P. davidiana* and hybrids as analyzed by principal component analysis. Sci. Hortic..

[67] De León-Zapata M.A., Pastrana-Castro L., Rua-Rodríguez M.L., Alvarez-Pérez O.B., Rodríguez-Herrera R., Aguilar C.N. (2016). Experimental protocol for the recovery and evaluation of bioactive compounds of tarbush against postharvest fruit fungi. Food Chem..

[68] Tesfay S.Z., Magwaza L.S. (2017). Evaluating the efficacy of moringa leaf extract, chitosan and carboxymethyl cellulose as edible coatings for enhancing quality and extending postharvest life of avocado (*Persea americana* Mill.) fruit. Food Packag. Shelf Life.

